# The emergence of *bla*_CTX-M-15_-carrying *Escherichia coli* of ST131 and new sequence types in Western China

**DOI:** 10.1186/1476-0711-12-35

**Published:** 2013-11-27

**Authors:** Lei Zhang, Xiaoju Lü, Zhiyong Zong

**Affiliations:** 1 Center of Infectious Diseases, West China Hospital, Sichuan University, Guoxuexiang 37, Chengdu 610041, China; 2 Division of Infectious Diseases, the State Key Laboratory of Biotherapy, Chengdu, China

**Keywords:** *Escherichia coli*, Plasmids, MLST, Beta-lactamases, Antimicrobial resistance

## Abstract

**Background:**

*bla*_CTX-M-15_, the most widely distributed gene encoding extended-spectrum β-lactamases globally, was not common in China. This study was performed to characterize *bla*_CTX-M-15_-carrying *Escherichia coli* in western China.

**Findings:**

Out of 144 *Escherichia coli* isolates from 20 hospitals in western China, 8 were found carrying *bla*_CTX-M-15_. *bla*_CTX-M-15_ was carried by isolates of ST131and 5 new STs (ST3342, ST3513, ST3516, ST3517 and ST3518). The 5 new STs shared 5 identical alleles out of 7 but only had up to 2 alleles identical to ST131. *bla*_CTX-M-15_ was located on plasmids of IncI1 (ST16) or IncFII-related group (four replicon types). The co-transfer of a few antimicrobial resistance genes including *qnrA*, *qnrB*, *qnrS*, *qepA*, *aac (6′)-Ib*-cr, *aac (3)-II*, *tetA*, *bla*_TEM_ and *bla*_OXA-1_ with *bla*_CTX-M-15_ were examined but only *bla*_TEM-1_ was found co-transferring with *bla*_CTX-M-15_.

**Conclusions:**

Five new STs of *E. coli* and three new types of IncFII-related plasmids carrying *bla*_CTX-M-15_ were identified. This study together with several reports suggested that *bla*_CTX-M-15_ has emerged in China and the interruption of both vertical and horizontal transmission of *bla*_CTX-M-15_ is required to hurdle its further spread.

## Findings

*Escherichia coli* producing extended-spectrum β-lactamases (ESBLs) is a challenge for clinical treatment and infection control. *bla*_CTX-M-15_ has emerged as the most widely distributed gene encoding ESBLs globally, but it was not common in China [1]. We performed a snapshot survey on the molecular epidemiology of *E. coli* carrying *bla*_CTX-M-15_ in Sichuan province, western China.

All non-duplicated *E. coli* clinical isolates (n = 144) that were recovered in 20 hospitals in Sichuan, western China, from June 22 to 25, 2011 were collected regardless of their antimicrobial susceptibility profiles and types of infections. The presence of *bla*_CTX-M_ genes were detected using both a single PCR with universal primers CTX-U1/U2 and a multiplex PCR [2,3] and 25.7% (37/144) isolates were found carrying *bla*_CTX-M_. A total of 17 isolates had a *bla*_CTX-M_ gene belonging to the *bla*_CTX-M-1_ group and the complete sequence of the *bla*_CTX-M-1_-like gene in all 17 isolates was obtained using PCR with primers ISEcp1IR-F and orf477-R [4]. Sequencing revealed *bla*_CTX-M-15_ in 8 isolates, corresponding to 21.6% of *bla*_CTX-M_ variants, and the remaining 9 isolates had either *bla*_CTX-M-55_ (n = 8) or *bla*_CTX-M-57_ (n = 1). The 8 isolates carrying *bla*_CTX-M-15_ were from urine (n = 5), sputum (n = 2) and ascites (Table 1). Although *bla*_CTX-M-15_ has become the dominant gene encoding ESBLs in many countries and *E. coli* of ST131 carrying *bla*_CTX-M-15_ was widely distributed [5], *bla*_CTX-M-15_ had only been occasionally found in isolates recovered before 2007 in China [1]. However, several studies on *E. coli* isolates obtained in 2007 and afterwards in China have found that *bla*_CTX-M-15_ accounted to 14% to 24% of *bla*_CTX-M_ variants detected from human [6,7]. The present study and previous reports therefore suggested the emergence of *E. coli* carrying *bla*_CTX-M-15_ in China in recent 5 years.

**Table 1:** **Molecular characteristics of 8 *E. coli *** **isolates carrying *bla***_**CTX-M-15**_

**Isolate**	**Source**	**ST**	**Phylogenetic group**	**Inc group**^**1**^	**pMLST/RST**^**1**^	**Additional resistance genes**^**3**^
A63	urine	3513	A	FIIA	F31:A-:B-	***bla***_**TEM**_**,***aac (3)-II*
H9	urine	3342	D	FIIA FIA FIB	F1:A2:B1	***bla***_**TEM**_**,***aac (3)-II*, *aac (6′)-Ib*-cr
D57	urine	3516	B2	FIIA FIA FIB	F1:A2:B20	***bla***_**TEM**_**,***aac (3)-II*
W19	urine	131	B2	FIIA	F2:A-:B-	***bla***_**TEM**_**,***qnrS*
V12	sputum	3518	B2	I1	1-ND^2^-10-ND^2^-6	
I10	sputum	3517	B2	I1	ST16 (1-5-10-8-6)	***bla***_**TEM**_**,***aac (3)-II*
U35	urine	131	B2	I1	ST16 (1-5-10-8-6)	*qnrS*
I20	ascites	131	B2	I1	ST16 (1-5-10-8-6)	*aac (3)-II*

^1^Inc, incompatibility; Inc group and pMLST/RST results are for plasmids carrying *bla*_CTX-M-15_.

The allele number of the IncI1 pMLST profile (*repI*, *ardA*, *trbA*, *sogS* and *pill*) is indicated.

^2^ND, not determined.

^3^Resistance genes that were co-transferred with *bla*_CTX-M-15_ are in bold.

Six of the isolates carrying *bla*_CTX-M-15_ belonged to the phylogenetic group B2 and the remaining two were of group A1 and D, respectively, determined as described previously [8] (Table 1). Using multi-locus sequence typing (MLST) [9], the 8 isolates carrying *bla*_CTX-M-15_ were assigned to 6 sequence types (ST) including ST131 and 5 new STs (Table 1). Of note, *E. coli* of ST131 was found carrying *bla*_CTX-M-3_, _-14_ and _-65_ but not _-15_ in our local settings previously [10]. This study demonstrated the emergence of the globally-spread ST131 carrying *bla*_CTX-M-15_ in our region. The 5 new STs (ST3342, ST3513, ST3516, ST3517 and ST3518) shared 5 identical alleles (*gyrB*, *icd*, *mdh*, *purA* and *recA*) out of 7 and these STs might therefore belong to a common clonal complex. In contrast, the 5 STs had only up to 2 alleles identical to ST131, suggesting the 5 STs and ST131 had different clonal origins.

Self-transmissible plasmids carrying *bla*_CTX-M-15_ were obtained from 7 out of the 8 isolates using mating as described previously [4]. The remaining isolate V12 did not yield transconjugants carrying *bla*_CTX-M-15_ despite repeated attempts but transformants carrying *bla*_CTX-M-15_ were obtained from V12 by electroporation with plasmids prepared using alkaline lysis [11]. This suggests that *bla*_CTX-M-15_ was carried by a non-conjugative plasmid in isolate V12. Plasmids carrying *bla*_CTX-M-15_ were prepared using alkaline lysis and were subjected to PCR-based replicon typing [12]. Four plasmids were of IncI1 and the other four belonged to the IncFII-related group, two of which contained replicons of IncFIA and IncFIB in addition to the IncFII-related replicon. IncF plasmid replicons sequence typing (RST) [13] and IncI1 plasmid MLST (pMLST) [14] were employed to investigate the relatedness of these plasmids carrying *bla*_CTX-M-15_ (Table 1). All of the IncI1 plasmids except the non-conjugative one from isolate V12 were of ST16 (1-5-10-8-6). According to the IncI1 pMLST database (http://pubmlst.org/plasmid/), ST16 IncI1 plasmids have been found carrying *bla*_CTX-M-15_ in isolates from cattle in the UK but were not found in human isolates carrying *bla*_CTX-M-15_ before. The identification of ST16 IncI1 plasmids in both cattle and human isolates suggested the transfer of *bla*_CTX-M-15_ between animal and human, which implicates that the control of the transmission of *bla*_CTX-M-15_ should address sources beyond human. Two alleles, *ardA* and *sogS*, both of which are associated with conjugation, were unable to be amplified from the non-conjugative plasmid (pV12) carrying *bla*_CTX-M-15_ from isolate V12. Therefore, the ST could not be assigned for pV12 but the remaining three alleles of pV12 were identical to those of ST16, suggesting that pV12 might be derived from a ST16 IncI1 plasmid. Four different RST profiles were present for the four IncF plasmids carrying *bla*_CTX-M-15_, suggesting that the spread of *bla*_CTX-M-15_ in our local settings might have been mediated by multiple IncF plasmids. Among the four RST types of IncF plasmids identified here, F2:A-:B- plasmids carrying *bla*_CTX-M-15_ appeared to be widely distributed and have been found in Canada, France, Italy and the UK (http://pubmlst.org/plasmid/), while the remaining three types, F1:A2:B1, F1:A2:B20 and F31:A-:B- have not been deposited in the RST database, representing new IncF RST types.

The co-transfer of a few antimicrobial resistance genes including *qnrA*, *qnrB*, *qnrS*, *qepA*, *aac(6′)-Ib*-cr, *aac(3)-II*, *tetA*, *bla*_TEM_ and *bla*_OXA-1_ with *bla*_CTX-M-15_ were examined for transconjugants and transformants by PCR. Unlike previous studies, the co-transfer of resistance genes with *bla*_CTX-M-15_ except for *bla*_TEM-1_ was not common in this study (Table 1).

In summary, although *bla*_CTX-M-14_ was the most common *bla*_CTX-M_ variant in China, the present study together with recent reports [6,7] suggested that *bla*_CTX-M-15_ has emerged in China. The prevalence of *bla*_CTX-M-15_-carrying isolates would compromise the efficacy of the widely-used broad-spectrum cephalosporins and therefore represents a serious challenge for clinical treatment and public health. The spread of *bla*_CTX-M-15_ in our local settings is mediated by two clonal complexes and by self-transmissible plasmids of IncI1 or IncF. The spread of isolates carrying *bla*_CTX-M-15_ in China warrants more studies. The interruption of both vertical and horizontal transmission of *bla*_CTX-M-15_ using infection control measures such as standard precautions and contact precautions appear to be the key to hurdle the further spread of this antimicrobial resistance determinant.

## Availability of supporting data

The data set supporting the results of this article is included within the article.

## Competing interests

The authors declare that they have no competing interests.

## Authors’ contributions

ZL carried out the study, participated in the sequence alignment and helped to draft the manuscript. LX participated in the design of the study and helped to draft the manuscript. ZZ conceived of the study, participated in the sequence alignment and coordination and drafted the manuscript. All authors read and approved the final manuscript.
